# Convergent accelerated evolution of mammal-specific conserved non-coding elements in hibernators

**DOI:** 10.1038/s41598-024-62455-8

**Published:** 2024-05-23

**Authors:** Daiki Nakayama, Takashi Makino

**Affiliations:** 1 Department of Biology, Faculty of Science, Tohoku University, 6-3, Aramaki Aza Aoba, Aoba-Ku, Sendai, 980-8578 Japan; 2 Graduate School of Life Sciences, Tohoku University, 6-3, Aramaki Aza Aoba, Aoba-Ku, Sendai, 980-8578 Japan

**Keywords:** Hibernation, Accelerated evolution, Conserved non-coding elements, Evolutionary genetics, Molecular evolution, Evolution, Genetics

## Abstract

Mammals maintain their body temperature, yet hibernators can temporarily lower their metabolic rate as an energy-saving strategy. It has been proposed that hibernators evolved independently from homeotherms, and it is possible that the convergent evolution of hibernation involved common genomic changes among hibernator-lineages. Since hibernation is a seasonal trait, the evolution of gene regulatory regions in response to changes in season may have been important for the acquisition of hibernation traits. High-frequency accumulation of mutations in conserved non-coding elements (CNEs) could, in principle, alter the expression of neighboring genes and thereby contribute to the acquisition of new traits. To address this possibility, we performed a comparative genomic analysis of mammals to identify accelerated CNEs commonly associated with hibernation. We found that accelerated CNEs are common to hibernator-lineages and could be involved with hibernation. We also found that common factors of genes that located near accelerated CNEs and are differentially expressed between normal and hibernation periods related to gene regulation and cell-fate determination. It suggests that the molecular mechanisms controlling hibernation have undergone convergent evolution. These results help broaden our understanding of the genetic adaptations that facilitated hibernation in mammals and may offer insights pertaining to stress responses and energy conservation.

## Introduction

Mammals have the ability to maintain their body temperature regardless of air temperature. However, some mammals experience low temperatures and/or scarcity of food or water on a seasonal basis, and such can cause a temporary reduction in metabolism and body temperature to conserve energy^1,2^. These mammals are called heterotherms. Hibernators are well-known examples of heterotherms that can reduce their body temperature by scaling back metabolism to a certain degree, and this reduction could be lethal to non-hibernators^3–7^. In addition, heterotherms can spontaneously raise their metabolic rate and body temperature from a low metabolic state^6,7^. Heterothermy is achieved through accurate control of body temperature, rather than being influenced by ambient temperatures, as seen in the brumation of ectotherms^1,3, 5–7^.

Heterotherms are distributed across several mammalian lineages, suggesting that heterotherms experienced convergent evolution. However, the ancestral states and evolutionary processes of these mammals remain unclear. Two conflicting hypotheses have been proposed to explain this evolution^8^. One hypothesis states that heterotherms evolved from an ectothermic ancestor before the emergence of homeotherms^9–13^. In fact, the first member of the mammalian lineage, echidna in *Monotremata*, exhibits an ectotherm-like change in body temperature^10,14^. However, most heterotherms can control their body temperature as can homeotherms. The other hypothesis is that heterothermy can be considered as an adaptive trait that originally evolved in homeotherms^15^. For our present study, we assumed that the latter hypothesis is accurate—i.e., that heterotherms evolved independently from homeotherms.

The evolution of heterothermy may be associated with a common genetic basis among heterotherms because some phenotypic convergences rely on common genetic backgrounds^16–19^. In addition, recent transcriptome analyses have consistently demonstrated that certain genes that are differentially expressed (i.e., DEGs) in response to changes in ambient temperature^20–28^. Therefore, searching for common nucleotide substitutions in regulatory regions among heterotherms may identify genomic regions associated with hibernation. Moreover, a productive approach for identifying these regulatory regions involves focusing on conserved non-coding elements (CNEs)^17,29–32^ because CNEs are widely conserved among species and therefore are likely to be cis-regulatory regions^33–36^. Thus, mutations in CNEs may be associated with changes in gene expression. Although the identification of CNEs relies on sequence conservation, it is plausible that some of the most functionally impactful CNEs also evolved under positive selection in particular lineages^37^. Therefore, investigating CNEs that have undergone accelerated accumulation of mutations—herein termed accelerated CNEs—is valuable for the identifying crucial regulatory regions involving lineage-specific traits^37–40^.

Based on this background, Ferris and Gregg (2019) hypothesized that, among hibernators, a common genetic background that included several accelerated CNEs drove the independent evolution of hibernation through changes in gene regulation. These investigators found that accelerated CNEs that were common in multiple hibernator lineages were located near genes associated with obesity susceptibility in humans^32^. Their findings were intriguing, but obesity is just one of the traits of hibernation. In addition, although they defined parallel accelerated CNEs as those that were accelerated in two or more lineages of four hibernator clades, using CNEs accelerated in a larger number of hibernator lineages allows us to investigate the generality for the evolution of hibernation among mammals. Furthermore, several transcriptome analyses of hibernators have inferred a set of candidate genes that respond to hibernation conditions^20–22, 24–28^, and this could bolster the identification of genes for which expression is altered under hibernating conditions. By integrating these findings with data on accelerated CNEs, the list of potential hibernation-related genes can be further refined. This study aimed to investigate the genetic commonalities associated with the capacity to hibernate by examining accelerated CNEs common to hibernator lineages and the genes of neighboring CNEs for which expression varies during hibernation.

## Materials and methods

### Hibernator and non-hibernator species

The UCSC multiple alignment format (MAF) file for 100 vertebrate assemblies in addition to the human genome (hg38 100-way) was downloaded to maximize the number of mammals included in our analysis. This file contains 62 mammalian assemblies from which we selected 50 non-hibernating and nine hibernating mammals. Daily torpor species (*Cricetulus griseus*, *Mus musculus*, *Rattus norvegicus*) were excluded from subsequent analyses. Hibernators were distributed across five different lineages (*Echinops telfairi*, *Elephantulus edwardii*, *Erinaceus europaeus*, bat, and rodent), and these species may have independently evolved the ability to hibernate^8,15^. In the bat lineage, the common ancestor of the three hibernating bats was considered a hibernator in our present study based on the assumption that the maximum parsimony was the most reasonable, i.e., that they may have evolved hibernation only a single time at the ancestral branch^41^. In the rodent lineage, it is unclear how hibernation evolved; therefore, we assumed that the rodent ancestor was also a hibernator. Therefore, we used the ancestral branch to test the acceleration of CNEs. Using MafFilter^42^, we constructed two MAFs containing 50 non-hibernators and 59 mammals.

### Identification of CNEs

From the 50 non-hibernator MAF, we identified conserved regions using phastCons^43^ provided by the open-source package PHAST (version1.5: http://compgen.cshl.edu/phast/downloads.php, with the following parameters: expected length = 45, target coverage = 0.3, rho = 0.31). From these conserved regions, we isolated coding regions that were annotated as coding sequences (refers to Ensembl 108 GRCh38.gff3) and extracted non-coding regions with length ≥ 20 bp as CNEs.

### Detection of non-coding accelerated regions among hibernators

For the CNEs, we computed p-values for acceleration across each of the specified branches (*E. telfairi*, *E. edwardii*, *E. europaeus*, bat, and rodent) using phyloP^44^ (with the following options: --method = LRT, --mode = ACC, --branch) and defined accelerated CNEs with a false-discovery rate threshold of 5%. Further, we computed the number of branches for which accelerated CNEs were identified in each region.

### Assignment of genes neighboring CNEs and gene ontology (GO) enrichment analysis

Genes were assigned to neighboring (within 1 Mb) CNEs using the GREAT method^45,46^. We then conducted GO enrichment analysis (biological process) using topGO^47^ (version 2.50.0: https://bioconductor.org/packages/release/bioc/html/topGO.html) to investigate the functional bias of genes neighboring accelerated CNEs compared with those neighboring total CNEs as the background. The target was set as the genes neighboring the accelerated CNEs in each hibernator lineage. The P-value was calculated using the weight01 method, and the false-discovery rate threshold was 5%.

### Identification of DEGs commonly associated with hibernation

To identify candidate genes commonly associated with hibernation across species, we integrated data from five studies that focused on differences in gene expression during normal and hibernation periods^22,24–27^. Further, genes reported to be differentially expressed in two or more studies were extracted, regardless of tissue and species.

## Results

### Accelerated CNEs shared among hibernator lineages

**Figure 1:**
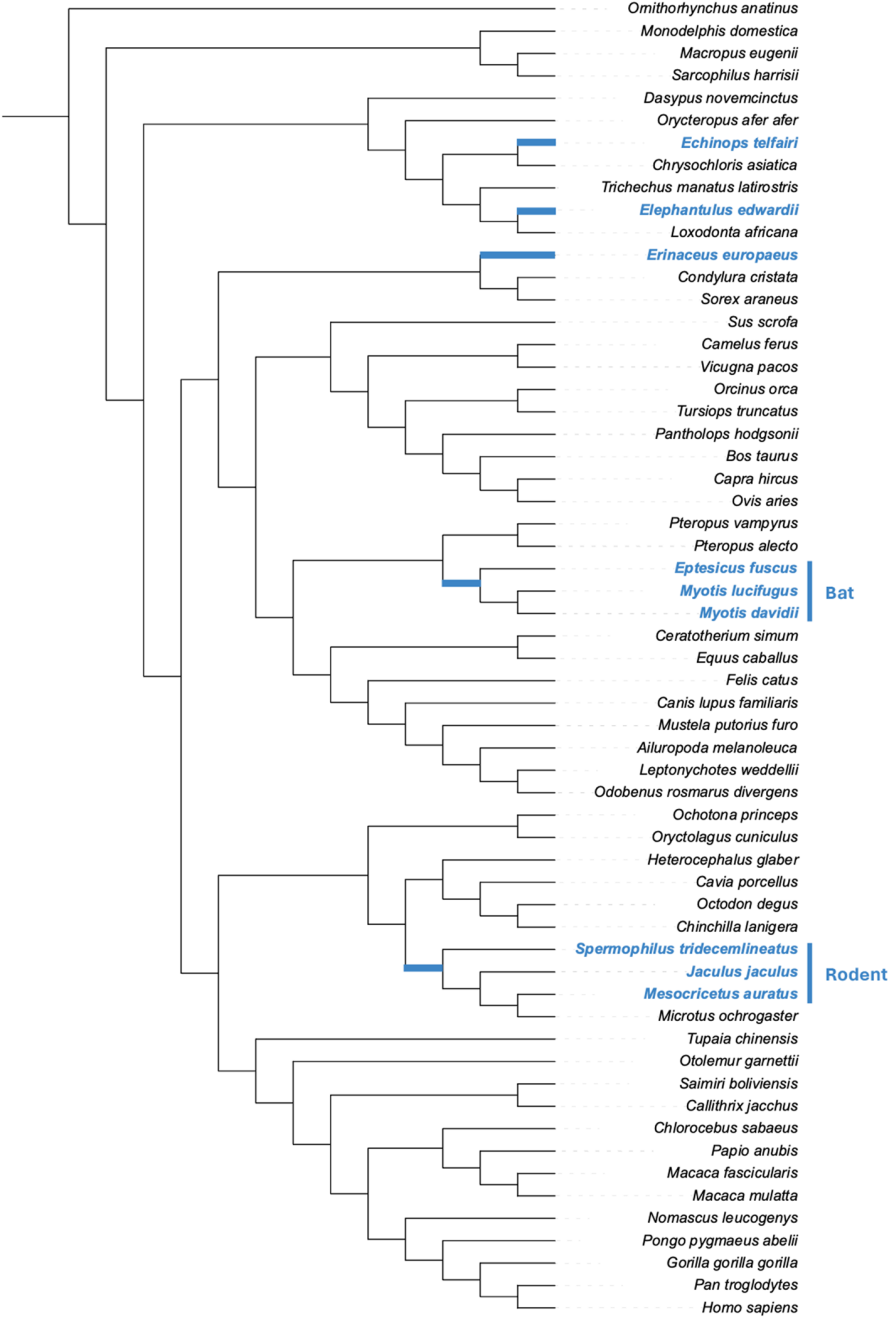
Species used in this study and the result of investigating accelerated CNEs. (**A**) This phylogenetic tree illustrates the relationships among the mammals used in this study. They were among the species used in the UCSC multiple alignment format (MAF) of 100 vertebrate assemblies with the human genome (hg38 100-way). The species colored in light blue are hibernators that are classified into five lineages: *Echinops telfairi*, *Elephantulus edwardii*, *Erinaceus europaeus*, bat, and rodent. The bat lineage comprises *Eptesicus fuscus*, *Myotis lucifugus*, and *Myotis davidii*, and the rodent lineage comprises *Spermophilus tridecemlineatus*, *Jaculus jaculus*, and *Mesocricetus auratus*, and accelerated evolution was detected at the branches colored in light blue.

To investigate overall CNEs among non-hibernators and identify accelerated CNEs in each hibernator lineage, we selected 50 non-hibernators and nine hibernators (*E. telfairi*, *E. edwardii*, *E. europaeus*, *Eptesicus fuscus*, *Myotis lucifugus*, *Myotis davidii*, *Spermophilus tridecemlineatus*, *Jaculus jaculus*, *Mesocricetus auratus*) from 100 vertebrates in the hg38 100-way UCSC multiple alignment (Fig. 1)^13^. Because *E. fuscus*,* M. lucifugus*, and* M. davidii* belong to the same clade, we named this as the bat lineage. Similarly, we named the clade comprising *S. tridecemlineatus*,* J. jaculus*, and* M. auratus* as the rodent lineage. The UCSC multiple alignment also contained three rodents (*M. musculus*, *R. norvegicus*, and *C. griseus*) that may be daily torpor species^13,48,49^. Because there is still some debate over the consideration of daily torpor and hibernation as continuous or discrete traits^13,50^, we excluded these three mammals to clearly observe the differences between hibernators and non-hibernators.

We identified 1,774,760 CNEs across 50 non-hibernators based on UCSC multiple alignments using PHAST. Further, their evolutionary acceleration in each of the five hibernator lineages was tested. As a result, 37,431, 46,507, 70,718, 38,765, and 18,971 accelerated CNEs were identified in the *E. telfairi*, *E. edwardii*, *E. europaeus*, bat, and rodent lineages, respectively. Moreover, we examined the number of accelerated CNEs shared between the lineages to investigate the commonality of accelerated evolution in hibernators. The lineage count was defined as the number of lineages. More than 12,000 accelerated CNEs were common to at least two hibernator lineages (Table 1). The number of accelerated CNEs shared among the hibernator lineages decreased with increasing lineage count, and only one CNE was accelerated in all hibernator lineages (Table 1).

**Table 1:** Number of accelerated CNEs.

Number of lineages sharing accelerated CNEs	Number of accelerated CNEs
2	12,528
3	714
4	26
5	1

### Screening candidate genes by examining DEGs under hibernating conditions

To narrow the number of candidate genes associated with hibernation, we used published data to examine differences in expression of the 3923 DEGs between normal and hibernation periods^22,24–27^. This revealed 1207, 42 and 6 DEGs that were located near the accelerated CNEs in ≥ 2, ≥ 3, and ≥ 4 hibernator lineages, respectively (Table 2).

**Table 2:** Overlap between DEGs and genes neighboring accelerated CNEs.

Number of lineages sharing accelerated CNEs	Number of overlapped genes
2	1207
3	142
4	6
5	0

### GO enrichment analyses of genes neighboring accelerated CNEs

#### Genes neighboring accelerated CNEs in multiple hibernator lineages

Further, we used GO enrichment analysis to focus on genes neighboring accelerated CNEs (within 1 Mb) in each lineage count to infer the functional bias of candidates associated with hibernation. A total of 172 terms exhibited statistically significant enrichment (false-discovery rate < 0.05) in the gene sets obtained from accelerated CNEs in two or more hibernator lineages (Table S1). Terms associated with transcriptional regulation and developmental processes were significantly enriched. Additionally, 16 terms were enriched in at least three hibernator lineages**,** whereas none were enriched in four or more hibernator lineages (Table S2).

#### DEGs neighboring accelerated CNEs in multiple hibernator lineages

To assess the functional bias of the genes screened as DEGs, we conducted a GO enrichment analysis for each lineage count. Two terms, namely ‘negative regulation of dendritic spine development’ and ‘endoplasmic reticulum tubular network organization’, were enriched in two or more hibernator lineages, whereas no terms were enriched in at least three hibernator lineages.

#### Genes accelerated in each hibernator lineage

Although our data thus far focused on the accelerated CNEs shared among hibernator lineages, variations in different regulatory regions or genes could result in similar phenotypes through gene pathway changes. To address this possibility, we performed a GO enrichment analysis for each hibernator lineage individually and examined the terms observed in all hibernator lineages. Statistically significant term enrichments were observed for each lineage (Table S3–7 ), and 18 terms, including regulation of transcription by RNA polymerase II, morphogenesis, and regulation of neuron differentiation, were shared among all hibernator lineages (Table 3).

**Table 3:** Relative enrichment of GO terms for genes located near accelerated CNEs shared among five hibernator lineages (Table S3–7).

GO.ID	Term
GO:0001525	Angiogenesis
GO:0007411	Axon guidance
GO:0001709	Cell-fate determination
GO:0000902	Cell morphogenesis
GO:0042733	Embryonic digit morphogenesis
GO:0035115	Embryonic forelimb morphogenesis
GO:0042472	Inner ear morphogenesis
GO:0050919	Negative chemotaxis
GO:0090090	Negative regulation of canonical Wnt signaling pathway
GO:0045665	Negative regulation of neuron differentiation
GO:0000122	Negative regulation of transcription by RNA polymerase II
GO:0048665	Neuron fate specification
GO:0001764	Neuron migration
GO:0010628	Positive regulation of gene expression
GO:0045666	Positive regulation of neuron differentiation
GO:0045944	Positive regulation of transcription by RNA polymerase II
GO:0009410	Response to xenobiotic stimulus
GO:0048485	Sympathetic nervous system development

## Discussion

**Figure 2:**
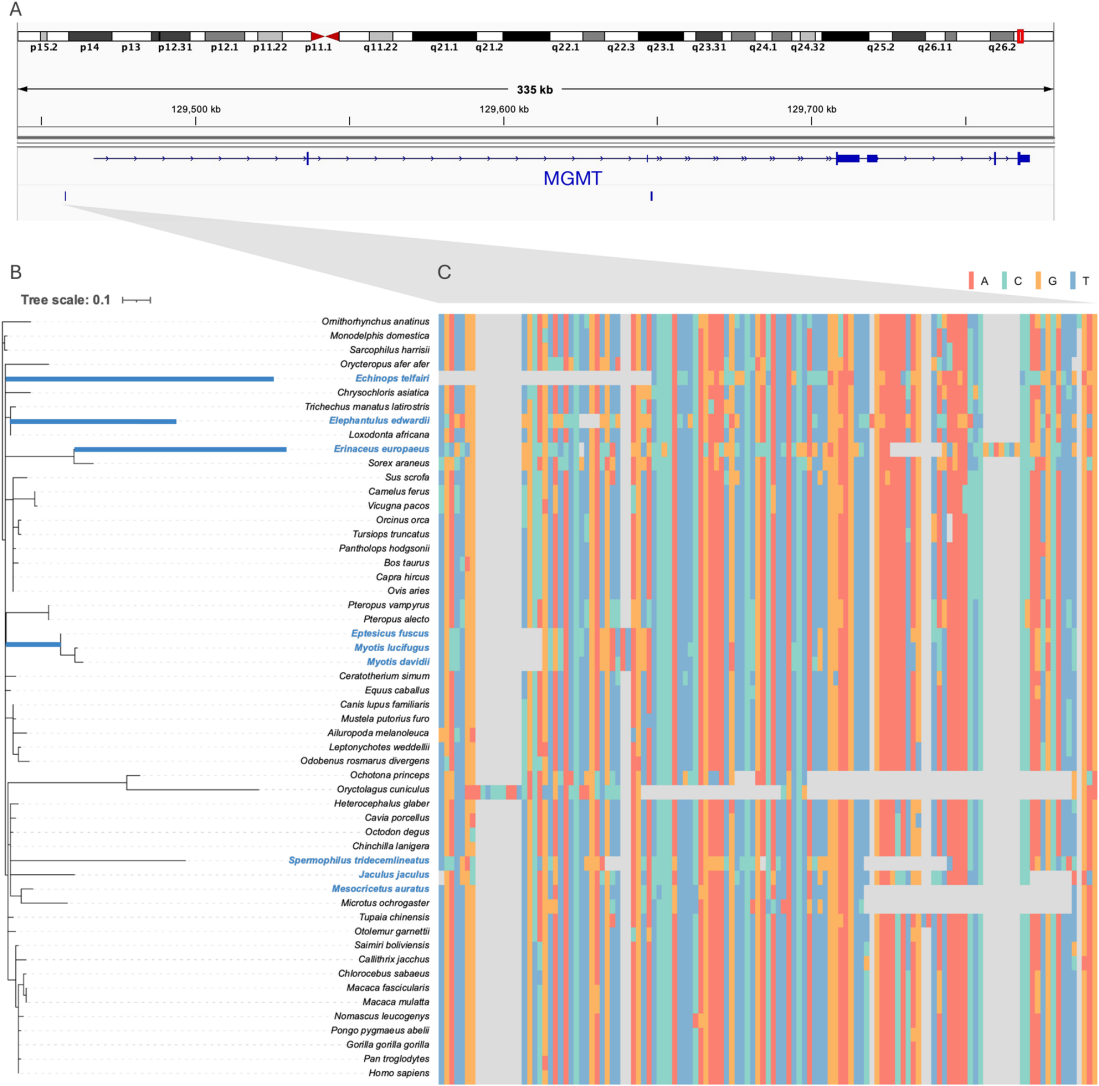
The accelerated CNE neighboring *MGMT* shared in all hibernator lineages. (**A**) The diagram show the exact location of the accelerated CNE shared among all hibernator lineages in human chromosome 10. (**B**) This evolutionary tree reflects the nucleotide substitution rates of CNEs neighboring *MGMT*, where accelerated evolution was observed in all five lineages. The species colored in light blue are hibernators. (**C**) This nucleotide alignment shows the accelerated CNE. The colors correspond to each nucleotide.

One accelerated CNE shared by all hibernator lineages was located approximately 10,000 bp upstream of *MGMT*, which encodes O6-methylguanine-DNA methyltransferase (Table 1). The evolutionary tree for that particular accelerated CNE indicated that accelerated evolution occurred in all hibernator lineages (Fig. 2). However, regarding the rodent clade, we expected that we would identify accelerated evolution at the ancestral node of rodents, but this tree indicated that the accelerated evolution occurred at descendant branches. This might suggest the limitation of phyloP to calculate the substitution rate at the ancestral branch. MGMT is involved in DNA repair and plays an important role in chemoresistance to alkylating agents^51^. To date, no study has suggested a relationship between *MGMT* and hibernation. However, other genes related to DNA repair are upregulated in the hypothalamus^26^. Likewise, the genome may be damaged during hibernation, and an accelerated CNE may upregulate *MGMT* and thereby contribute to DNA repair and genome protection throughout hibernation.

**Figure 3:**
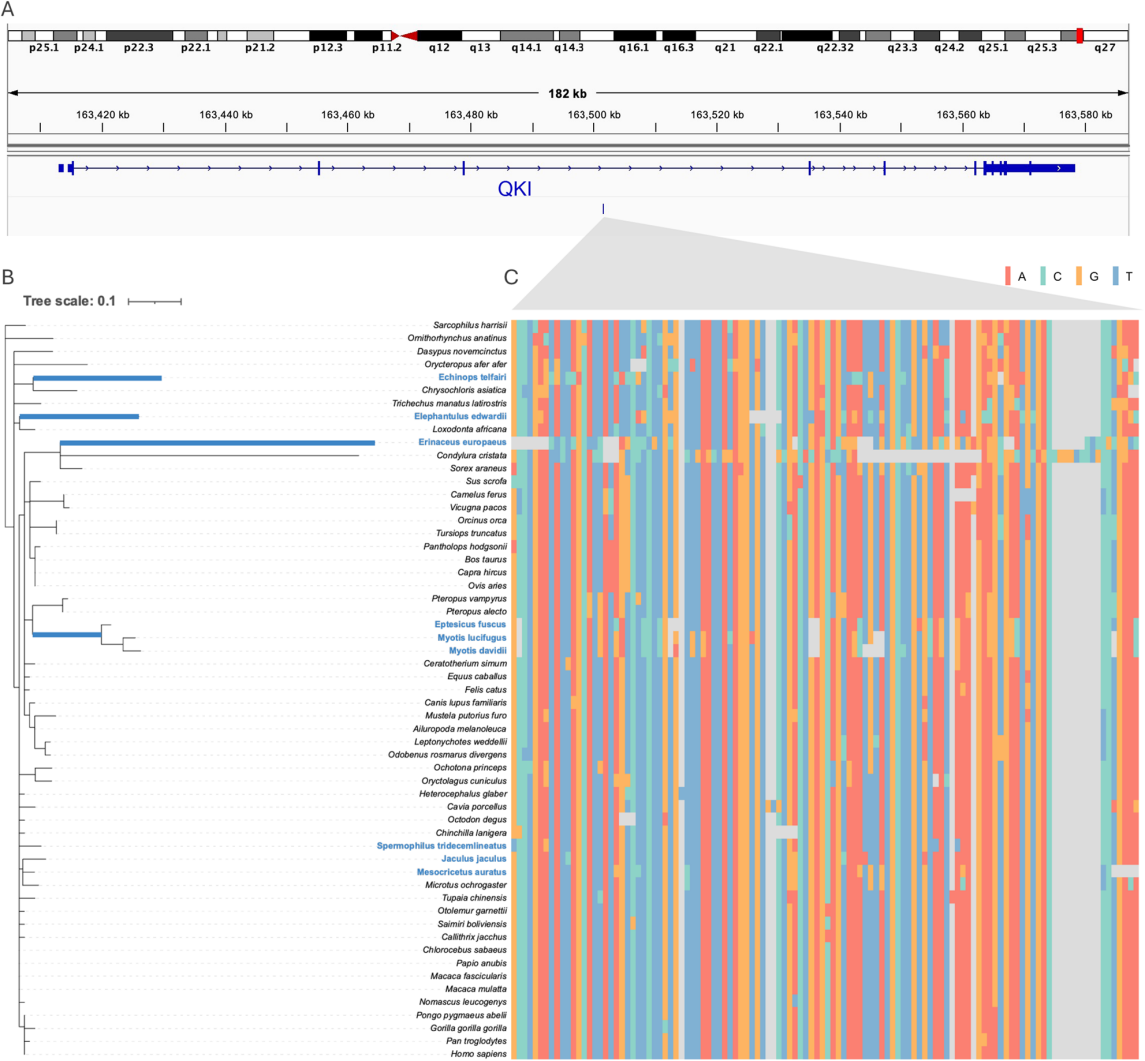
The accelerated CNE located in the intron of *QKI*. (**A**) The diagram shows the exact location of the accelerated CNE shared among four hibernator lineages in human chromosome 6. (**B**) This evolutionary tree shows accelerated evolution observed in four lineages, excluding the rodent lineage, for a CNE neighboring *QKI.* The length of the branches in the phylogenetic tree indicates the nucleotide substitution rate. The species colored in light blue are hibernators. (**C**) This nucleotide alignment shows the accelerated CNE. The colors correspond to each nucleotide.

We found *QKI*, a DEG, that is known to be cooperated with heterothermy (Table 2). The evolutionary tree supported that the CNE located in the intron of *QKI* was accelerated in four hibernator lineages (Fig. 3). *QKI* encodes an RNA-binding protein enriched in the central nervous system and heart during embryonic development and adulthood of human and mouse^52^. *QKI* encodes three major isoforms: QKI-5, -6, and -7. *QKI*-5 is abundantly expressed in heart tissue, and both *QKI*-5 and *QKI*-6 can inhibit ischemia/reperfusion-induced apoptosis of cardiomyocytes^53–55^. In addition, *QKI* is upregulated during hibernation in the brain of bats^24^ and the heart of ground squirrels^22^. Hibernators reduce their metabolism and heart rate during hibernation, which causes ischemia and reperfusion. Thus, *QKI* may contribute significantly to countering ischemia caused during hibernation in the heart. Nevertheless, an accelerated CNE may contribute to changes in *QKI* expression during hibernation.

GO enrichment analysis of genes located near the accelerated CNEs common to multiple hibernator lineages yielded significant terms only when CNEs accelerated in two or three lineages were used (Tables S1 and S2). GO enrichment analysis using genes located near the accelerated CNEs in each lineage revealed 18 terms common across all lineages (Table 3). This indicated that regions that experienced accelerated evolution had certain common aspects, suggesting that similar genetic factors may give rise to the evolution of each hibernator. Common terms were associated with changes in gene regulation, morphogenesis, and cell fate, indicating potential cooperation between mechanisms of accelerated evolution. Although there were no enriched GO terms after narrowing the number of CNEs with the inclusion of more lineages, common enriched GO terms were found in each hibernator lineage. This may imply that different genes in the same molecular pathways or regulatory networks had undergone functional convergence.

We excluded daily torpor species from the analyses because rodents are the only such species for which genome sequences are available, despite the fact that several other mammals are daily torpor species. It is important that the target species be present in different clades of mammals because this analysis searched for genetic commonalities among all mammals. Therefore, the inclusion of daily torpor rodents in the analysis could have confounded the comparison with mammalian hibernators. In addition, hibernators, daily torpor species, and non-hibernators are mixed among rodents, and their evolutionary processes must be very complex. Thus, daily torpor species were excluded to ensure that we could clearly differentiate between hibernating and non-hibernating species.

This study serves as an extension of the report published by Ferris and Gregg, incorporating more extensive datasets for the inclusion of more species and identifying common genetic features among mammalian hibernators. By using new genomic data, our analysis adheres to the established pipeline for CNE detection and extends its application to a broader phylogenetic spectrum. Interestingly, through the foundational analysis pipeline and parameters that remained consistent with the Ferris and Gregg study, there was only 25% concordance between the accelerated CNEs identified in our study and those reported by Ferris and Gregg. As to why the overlap with accelerated CNE detected by Ferris and Gregg is not high, the number of species that were included in the analysis might be considered. A total of 50 non-hibernator genomes were used to identify CNEs in our present study, but 14 non-hibernator genomes were used in the Ferris and Gregg study. This suggests that the inclusion of additional species can significantly enhance the identification of genomic elements. Additionally, we changed the length of CNEs from 50 to 20 bp to obtain more candidate regions because we narrowed the number of CNEs by examining the number of lineages that shared accelerated CNEs among hibernator lineages in later analyses. This change also might have affected the number of CNEs identified.

One strength of our study is that we utilized DEGs to differentiate between genes involved in hibernation and all genes in our analysis. In addition, we performed GO enrichment analyses to infer biological signatures of genes neighboring the accelerated CNEs that are potentially involved in hibernation. One limitation of our study is that we did not investigate whether the CNEs we identified indeed contribute to the regulation of neighboring genes. Moreover, we assumed that heterotherms evolved independently from homeotherms. If we could confirm that genes regulated by the accelerated CNEs have a critical relation with hibernation, we will be able to determine the ancestral state in detail by analyzing the evolutionary processes of these genes.

## Conclusion

Our findings reveal a subset of genes adjacent to the accelerated CNEs that are potentially implicated in gene repair and tolerance to ischemia, both of which are integral to the hibernation phenotype. Additionally, the expression levels of genes neighboring accelerated CNEs were used to identify genes that may be involved in hibernation from those obtained in the analysis. By correlating accelerated CNEs with gene-expression profiles, we provide insights into the genomic adaptations that underlie hibernation. These results not only enhance our understanding of hibernation but also contribute to the broader discourse concerning the evolutionary processes shaping this complex trait.

### Supplementary Information

Supplementary Tables.

## Data Availability

Raw data are available through the Dryad Digital Repository (https://datadryad.org/stash/share/-yHoXxJWnHbNO_3F1LNYCCt1tXvVAlQa3Un9Dm4o-to). The multiple alignment format of 100 vertebrate assemblies with the human genome was downloaded from UCSC (https://hgdownload.soe.ucsc.edu/goldenPath/hg38/multiz100way/maf/). The method phastCons was executed using phastcons100way.mod (https://hgdownload.soe.ucsc.edu/goldenPath/hg38/phastCons100way/), and phyloP was executed using phyloP100way.mod (https://hgdownload.soe.ucsc.edu/goldenPath/hg38/phyloP100way/).

## Abbreviations

CNEs: Conserved non-coding elements
DEGs: Differentially expressed genes
GO: Gene ontology
MAF: Multiple alignment format
